# Attitudes Toward Coercion Among Mental Healthcare Workers in Italy: A Cross-Sectional Study

**DOI:** 10.3390/healthcare13141680

**Published:** 2025-07-12

**Authors:** Calogero Gugliotta, Antonino Amato, Giuliano Anastasi, Teresa Rea, Roberto Latina, Pasquale Iozzo, Stefano Bambi

**Affiliations:** 1 Nursing and Midwifery Health Professions Unit, Provincial Health Authority of Palermo, 90141 Palermo, Italy; calogerogugliotta@asppalermo.org (C.G.); antoninoamato@asppalermo.org (A.A.); 2 Department of Biomedicine and Prevention, University of Rome Tor Vergata, 00133 Rome, Italy; pasquale.iozzo@students.uniroma2.eu; 3 Centro d’Eccellenza Mediterraneo per lo Sviluppo Accademico della Ricerca Infermieristica (CEMSIR), 80138 Naples, Italy; teresa.rea@unina.it (T.R.); roberto.latina@unipa.it (R.L.); 4 Department of Public Health, University of Naples “Federico II”, 80138 Naples, Italy; 5 Department of Health Promotion, Mother and Childcare, Internal Medicine and Medical Specialties (PROMISE), University of Palermo, 90127 Palermo, Italy; 6 Department of Health Sciences, University of Florence, 50139 Florence, Italy; stefano.bambi@unifi.it; 7 Research and Development of Clinical Practice Unit, Careggi University Hospital, 50134 Florence, Italy

**Keywords:** mental health, coercion, attitudes, healthcare workers, nursing, Italy, SACS

## Abstract

**Background/Objectives**: Coercive measures remain a common practice in mental health, despite ethical concerns, potential risks, and uncertain efficacy. Mental healthcare workers’ (MHCWs) attitudes toward coercion can influence their use. However, research in Italy is limited. This study aimed to investigate Italian MHCWs’ attitudes toward coercion and their associations with sociodemographic and professional characteristics. **Methods**: A cross-sectional study was conducted on 356 MHCWs from a mental health department in Southern Italy. Participants completed the Staff Attitude to Coercion Scale (SACS), which assesses negative, pragmatic, and positive attitudes toward coercion. Descriptive statistics (i.e., frequencies, percentages, means) and bivariate analyses (i.e., one-way ANOVA) were used to explore the associations between variables. **Results**: The majority of participants were male (56.7%), nurses (50.3%), and worked in acute psychiatric settings (52%), with a mean age of 51.08 years (±10.59) and 13.74 years (±12.14) of experience in mental health. Attitudes differed significantly according to age, sex, professional role, and work setting. More negative attitudes were found among staff in residential settings and non-caring roles (*p* < 0.001). Pragmatic attitudes were lower among older staff (*p* = 0.012) and among those in residential settings and non-caring roles (*p* < 0.001). Positive attitudes were higher among males (*p* = 0.001), nursing staff (*p* < 0.001), and staff in acute settings (*p* = 0.049). **Conclusions**: Italian MHCWs reported different attitudes toward coercion, which was influenced by personal and professional factors. These findings highlight the need for targeted interventions and policy strategies to promote attitudinal change, particularly in settings where positive attitudes are prevalent.

## 1. Introduction

Coercion remains one of the most controversial and debated practices in contemporary mental healthcare [1]. Its definition varies widely across theoretical frameworks and cultural contexts and often lacks clear boundaries [2,3]. The Swiss Academy of Medical Sciences defines coercion as any intervention conducted against the patient’s will or without consent [4]. According to the World Health Organization, coercion encompasses a wide range of practices, including physical and chemical restraint, seclusion, involuntary hospitalization, and forced treatment [5].

Although coercion may be deemed necessary to ensure safety [2], its use raises significant ethical and legal concerns. These include potential violations of patient autonomy, breach of human rights, and damage to therapeutic relationships [6,7]. Furthermore, coercion is associated with relevant physical and psychological harm [8,9], and evidence supporting its clinical effectiveness remains inconclusive [10]. Consequently, its use in mental healthcare has been increasingly questioned [11].

In response to these concerns, international organizations [5,12] and scholars [13] have advocated for a reduction in coercive measures. Nevertheless, coercion is widespread in mental health systems worldwide [14]. Recent meta-analyses have reported prevalence rates of 14.4% for physical restraint, 15.8% for seclusion, and 25.7% for chemical restraint [15], while the feasibility of eliminating such interventions continues to be argued [16].

Mental healthcare workers (MHCWs) play a central role in the application of coercive measures, often balancing ethical dilemmas, patient safety, and institutional expectations [17,18]. However, research has consistently highlighted ambivalent attitudes toward coercion [19,20]. While MHCWs acknowledge the ethical challenges of coercion, they often perceive it as necessary [21], beneficial [22], or a routine intervention in clinical practice [23]. Moreover, some studies suggest that coercion may also reflect underlying power dynamics within psychiatric settings [3], being used to enforce compliance [24], maintain control [25], or even as a form of punishment [19]. Lastly, the social context of care can affect the therapeutic alliance, the de-escalation of aggression, and the use of coercion [26,27,28]. This complex interplay of factors may contribute to the normalization of coercive practices [29] and limited motivation to reduce their use [30].

Indeed, recent literature has emphasized the multifactorial nature of decision making related to coercion, involving organizational, contextual, and individual variables [29,31,32,33]. Among these, MHCWs’ attitudes have received growing attention [20,29,34], as they are presumed to influence the use of coercion [34,35,36], consistent with psychological theories on attitude-driven behavior [37]. However, evidence on whether attitudes predict the actual use of coercion is mixed [34]. While some studies support a relationship between attitudes and behavior [29,36,38,39], others do not find significant associations [35,40].

Additionally, research suggests that attitudes toward coercion are shaped by personal characteristics [20,21,34,40,41], a notion supported by behavioral theories [42]. Variables such as age, sex, professional role, clinical experience, education, and work setting appear to be involved in this process [29,30,31,35,38,43,44,45]. However, these findings remain unclear, underscoring the need for further research [20,34,46].

In Italy, despite the growing discussion on the use of coercion in psychiatric settings [47,48], data on staff attitudes remain limited [49]. This gap is particularly worrying, given the persistence of coercive practices within Italian mental health services [50]. To our knowledge, no study has systematically explored how sociodemographic and professional characteristics relate to MHCWs’ attitudes toward coercion in the Italian context.

Therefore, this study aims to describe the attitudes of Italian MHCWs toward coercion and their associations with sociodemographic and professional characteristics.

## 2. Materials and Methods

### 2.1. Study Design

This cross-sectional study was conducted as part of an institutional quality improvement project aimed at reducing the use of coercive practices within the Department of Mental Health of the Provincial Health Authority of Palermo, Sicily, Italy. To ensure methodological rigor and transparency, the study adhered to the Strengthening the Reporting of Observational Studies in Epidemiology (STROBE) guidelines (see Appendix A). The research questions that guided the study were: What are the attitudes toward coercion among Italian MHCWs? What are the variables that influence Italian MHCWs’ attitudes toward coercion?

### 2.2. Sample and Setting

In the Italian National Health Service, 26,276 MHCWs are employed in the Departments of Mental Health working in different facilities, including Community Mental Health Centers (CMHCs), General Hospital Psychiatric Units (GHPUs), Day Care Facilities (DCFs), and Residential Facilities (RFs) [51]. CMHCs provide outpatient care and community-based psychiatric services; GHPUs deliver acute inpatient psychiatric treatment within general hospitals; DCFs offer short- and medium-term rehabilitative care in semi-residential settings; and RFs provide long-term residential care for individuals with severe mental disorders [52]. This study was conducted within the Department of Mental Health of the Provincial Health Authority, comprising nine CMHCs, nine DCFs, eight GHPUs, and five RFs across the province. The GHPUs were located within general hospital buildings, primarily on the main floor (62.5%). Each unit had patient rooms with toilets, common areas (i.e., a living room with television), and designated smoking areas. No units had access to outdoor spaces. All units admitted both voluntary and involuntary patients, were routinely locked, and allowed family visits on designated hours.

Considering that all facilities within the Department of Mental Health may implement some form of coercion (e.g., forced treatment) and that staff members may rotate between settings to ensure service coverage (e.g., during staff shortages), all the Department’s facilities and MHCWs were included in the study. Participants were recruited using non-probabilistic convenience sampling. The inclusion criteria were: (1) being a mental healthcare worker (e.g., nurses, nursing aides, psychiatrists); (2) aged 18 years or older; (3) fluent in Italian; and (4) able and willing to provide informed consent. Individuals who were not directly involved in patient care (e.g., administrative or cleaning staff) and those absent from work for over one year were excluded. The required sample size was calculated using Cochran’s formula [53], using a 95% confidence interval, a 5% margin of error, and an assumed prevalence of 50% to maximize the sample size according to the World Health Organization guidelines and established methodologies [53,54]. Therefore, the required sample size is 384 participants.

### 2.3. Data Collection and Questionnaire

Data were collected between May 2023 and December 2023 using a structured, self-administered questionnaire. Paper copies of the questionnaire and accompanying materials were distributed with the assistance of a head nurse at each participating facility. Each potential participant received a cover letter explaining the purpose and procedures of the study, an informed consent form, and a questionnaire. Participation was voluntary and conditional on the provision of written informed consent. Each respondent was assigned a unique identification code to ensure confidentiality.

The questionnaire included 21 closed-ended items, organized into two sections. The first section gathered sociodemographic and professional information, including sex (male or female), age (in years), professional role (i.e., nurses, nursing aides, psychiatrists), work setting (Community Mental Health Centers, General Hospital Psychiatric Units, Day Care Facilities, or Residential Facilities), years of work experience, and years of mental health experience. The second section assessed attitudes toward coercion using the Staff Attitude to Coercion Scale (SACS) [55], a validated instrument comprising 15 statements concerning the use of coercion and related beliefs. Respondents rated each item on a 5-point Likert scale, ranging from 1 (“strongly disagree”) to 5 (“strongly agree”), with a total score ranging from 15 to 75 [55]. The SACS evaluates three dimensions of attitude: negative (coercion as offensive), pragmatic (coercion as care and security), and positive (coercion as treatment) [55].

No validated Italian version of the SACS was available at the time of data collection. Therefore, a rigorous cross-cultural adaptation process was performed following the methodology proposed by Beaton et al. [56]. The translation committee included the study authors and four bilingual translators (two native Italian speakers and two native English speakers, fluent in both languages). Cultural and linguistic equivalence was achieved with strong inter-rater agreement (k = 0.85) [57]. Face and content validity were assessed by a panel of 15 experts, comprising three head nurses and four registered nurses from mental health settings, two nursing professors, and five nurse researchers experienced in instrument validation, following established methodologies [58,59]. The Content Validity Ratio (CVR) ranged from 0.65 to 1.0, exceeding the minimum acceptable threshold of 0.49 [59]. The Item-Level Content Validity Index (I-CVI) ranged from 0.76 to 1.0, surpassing the recommended cut-off of 0.70 [60]. The Scale-Level Content Validity Index (S-CVI/Ave) was 0.80, which met the minimum recommended standard [58]. Expert panel feedback also confirmed the instrument’s clarity, readability, and conceptual consistency [61]. Therefore, the translated version was deemed appropriate for use in this study.

After data collection, Venturini et al. [62] published a validated Italian version of the SACS, demonstrating acceptable psychometric properties, with Cronbach’s alpha values ranging from 0.64 to 0.77 across the three subscales. A comparison between our translated version and that of Venturini et al. revealed a comparable item structure. Consequently, for the analysis, the data were scored according to the validated Italian version [62]. Considering the absence of established cut-offs and in line with the literature [55], subscale scores were calculated as the mean of the item responses within each dimension, with higher scores reflecting stronger attitudes.

### 2.4. Data Analysis

Descriptive and inferential statistics were used to analyze the data. Frequencies, percentages, medians, means (Ms), and standard deviations (SDs) were calculated for all variables. The assumptions of normality and homogeneity of variance were assessed using the Shapiro–Wilk and Levene’s tests, respectively, confirming the appropriateness of parametric analyses. Associations between continuous variables and SACS scores were evaluated using Pearson’s correlation or Spearman’s rho, depending on the distribution of the data. Group differences in attitudes were analyzed using independent sample *t*-tests for two-group comparisons and one-way ANOVA for comparisons involving three or more groups. When ANOVA results were statistically significant, Tukey’s post-hoc tests were conducted to identify specific group differences. To address potential confounding variables, stratified analyses were conducted across relevant sociodemographic subgroups. Missing data were excluded from the analysis. All statistical analyses were performed using IBM SPSS© (Statistical Package for the Social Sciences) version 26. Statistical significance was set at *p* < 0.05.

### 2.5. Ethical Considerations

The study was approved by the Administration of the Provincial Health Authority of Palermo (protocol No. 118014/2023, dated 3 May 2023). The study was conducted according to the ethical principles outlined in the Declaration of Helsinki, Good Clinical Practice Guidelines, and relevant Italian legal and research ethics requirements for non-interventional studies. As this study did not involve patients, no personal clinical data were collected. All participants were fully informed about the study’s objectives and procedures and their right to withdraw at any time without consequences. Participation was voluntary, and written informed consent was obtained in compliance with Italian Legislative Decree no. 196 of 30 June 2003. Participants’ confidentiality was strictly maintained. All data were anonymized before analysis and stored securely with access limited to the research team.

## 3. Results

### 3.1. Sociodemographic and Professional Characteristics

A total of 401 MHCWs were recruited in this study, and 367 responded. After excluding 11 questionnaires due to missing or incomplete responses, the final sample comprised 356 participants. The majority were male (n = 202, 56.7%), with a mean age of 51.1 years (SD ± 10.6). On average, participants reported 22.61 years (SD ± 12.81) of total work experience, including 13.74 years (SD ± 12.14) specifically in the mental health settings. Nurses represented the largest professional group (n = 179, 50.3%), and more than half of the respondents worked in psychiatric units (n = 185, 52.0%). A detailed overview of participants’ sociodemographic and professional characteristics is provided in Table 1.

### 3.2. Attitudes Toward Coercion

Participants reported moderately negative attitudes (M = 3.06, SD ± 0.79), more pronounced pragmatic attitudes (M = 3.33, SD ± 0.67), and relatively lower positive attitudes (M = 2.70, SD ± 0.71) toward coercion. The highest levels of agreement were observed for items suggesting that coercion is sometimes necessary when dealing with dangerous or aggressive patients (M = 4.04, SD ± 0.99) and dangerous situations (M = 3.93, SD ± 0.96). In contrast, respondents expressed low agreement with the notion that more coercion should be used in the treatment (M = 2.00, SD ± 0.97). Further insights into these attitudes, reported per item and subscale, are presented in Table 2.

### 3.3. Associations and Differences in Attitudes by Sociodemographic and Professional Characteristics

To explore how MHCWs’ attitudes toward coercion varied according to sociodemographic and professional characteristics, correlational analyses (Table 3) and group comparisons (Table 4) were performed. The results indicate that attitudes toward coercion were significantly associated with age, sex, professional roles, and work setting. In contrast, neither overall work experience nor length of mental health experience was significantly associated with attitudes.

Correlational analyses revealed a slight but significant inverse relationship between age and pragmatic attitudes (r = −0.128, *p* ≤ 0.05). Group comparisons further confirmed age-related differences in pragmatic attitudes (*p* = 0.012). Participants aged 60 years and older reported significantly lower pragmatic attitudes (M = 3.14, SD ± 0.72) than those aged 40–49 (M = 3.45, SD ± 0.67; *p* = 0.022) and 50–59 years (M = 3.39, SD ± 0.58; *p* = 0.029). However, no significant differences were observed between the participants aged ≥ 60 years and those aged <40 years (M = 3.34, SD ± 0.73; *p* = 0.364). No significant age-related differences were found for negative or positive attitudes.

Regarding sex, no significant differences emerged for negative or pragmatic attitudes. However, male participants expressed significantly more positive attitudes toward coercion (M = 2.80, SD ± 0.74) than female participants (M = 2.56, SD ± 0.64; *p* = 0.001).

Significant differences were also found across professional roles in all three attitudinal dimensions (*p* < 0.001). Negative attitudes were higher among other healthcare professionals (e.g., social workers and psychologists) (M = 3.90, SD ± 0.76), significantly exceeding those of psychiatrists (M = 3.18, SD ± 0.74; *p* = 0.001), nursing aides (M = 3.02, SD ± 0.72; *p* < 0.001), and nurses (M = 2.93, SD ± 0.78; *p* < 0.001). Pragmatic attitudes were the lowest among other professionals (M = 2.72, SD ± 0.73), significantly lower than those of psychiatrists (M = 3.30, SD ± 0.80; *p* = 0.002), nursing aides (M = 3.38, SD ± 0.61; *p* < 0.001), and nurses (M = 3.39, SD ± 0.62; *p* < 0.001). Negative attitudes were higher among nursing aides (M = 3.00, SD ± 0.75) and significantly greater than those of nurses (M = 2.70, SD ± 0.65; *p* = 0.003), psychiatrists (M = 2.40, SD ± 0.56; *p* < 0.001), and other professionals (M = 2.11, SD ± 0.54; *p* < 0.001).

Attitudes also varied significantly according to the work setting. Differences were found in negative, pragmatic (*p* < 0.001), and positive attitudes (*p* = 0.049). Staff working in residential settings reported the highest levels of negative attitudes (M = 3.53, SD ± 0.80), which were significantly greater than those in community (M = 3.21, SD ± 0.70; *p* = 0.014) and acute settings (M = 2.78, SD ± 0.71; *p* < 0.001). Conversely, their pragmatic attitudes were the lowest (M = 3.00, SD ± 0.71), significantly below those of professionals in community (M = 3.28, SD ± 0.70; *p* = 0.014) and acute settings (M = 3.50, SD ± 0.58; *p* < 0.001). Regarding positive attitudes, staff in acute settings reported higher scores (M = 2.78, SD ± 0.70) than those in community settings (M = 2.56, SD ± 0.60; *p* = 0.046), while no significant difference emerged between acute and residential settings (M = 2.68, SD ± 0.80; *p* = 0.809).

## 4. Discussion

This study describes the attitudes of MHCWs toward coercion and explored their associations with key sociodemographic and professional variables in the Italian context. To the best of our knowledge, this is the largest empirical investigation of this topic in Italy, offering important insights into how professionals perceive and justify the use of coercion. Despite extensive psychiatric reforms in Italy, including the closure of asylums and the shift to community-based care [63], coercive practices remain widespread [50], underscoring the relevance of this research.

Overall, participants expressed moderately negative attitudes, more pronounced pragmatic attitudes, and relatively low positive attitudes toward coercion. This attitudinal pattern is consistent with international findings [20,34,64], suggesting that although Italian MHCWs are aware of ethical challenges, they often regard coercion as an unavoidable component of clinical practice [17,20,21,22,23,29,41].

Significant differences in attitudes emerged based on age, sex, professional role, and work setting, while neither total work experience nor experience in mental healthcare showed significant associations. This contrasts with earlier studies suggesting that professional experience influences attitudes [21,38,43,45,65], suggesting that in the Italian context, other variables may play a more critical role.

Age was inversely associated with pragmatic attitudes, with significantly lower scores reported by older professionals. This finding is consistent with previous research [66] and may reflect generational differences in values, ethical reasoning, or exposure to systemic changes in psychiatric care. The literature suggests that older age is associated with greater compassion, empathy, and ethical sensitivity [67,68], factors which may increase awareness of the ethical challenges inherent in the use of coercion [69]. In addition, older professionals may have directly experienced Italy’s historical transition toward rights-based and deinstitutionalized mental health care [63], potentially fostering a more cautious view of coercive practices. However, it should be noted that the observed differences, although statistically significant, were modest in magnitude. Furthermore, some studies have found no significant association between age and attitudes toward coercion [21]. These considerations suggest that, while age could play a role, its influence might be limited, and further research is needed to better clarify this relationship.

Sex also emerged as a significant factor, with male participants reporting significantly more positive attitudes toward coercion. This finding is consistent with previous research indicating that male staff members are more likely to perceive coercion as appropriate [29,43,66] and are more frequently involved in its application [44,70]. A plausible explanation lies in differential exposure to violence: male staff members are more involved in aggression [71], as violent incidents are mostly perpetrated by male patients [72] and tend to target same-sex staff members [73]. This may reinforce the perception of coercion as a protective measure. Moreover, social role theory suggests that gender norms influence behavior [74], a dynamic that is evident in mental health settings [75]. Studies have shown that male professionals are often assigned to manage aggressive patients, a role that may reinforce professional identity and foster a sense of validation [76]. This process may contribute to more favorable attitudes toward coercion among male MHCWs, reflecting the gendered dynamics in mental health settings. Nevertheless, it is important to recognize that although statistically significant, the differences were relatively small. Additionally, some previous studies reported no significant sex differences [21]. Consequently, while sex may influence attitudes toward coercion, its practical relevance remains elusive, highlighting the need for further investigation.

Professional roles were significantly associated with attitudes toward coercion. Nurses and nursing aides were more likely to endorse coercion as a therapeutic tool, which is consistent with previous findings [21,35,41,77]. In contrast, professionals such as psychologists and social workers expressed stronger negative attitudes and were less likely to endorse pragmatic or positive views of coercion, a trend also reported in earlier research [64]. These differences may stem from varying degrees of involvement in coercive practices [35]. Nurses and nursing aides are typically more directly involved in coercive measures. Over time, frequent exposure may lead to the normalization of such interventions, contributing to more accepting or even justificatory views [46,66,69,78]. In contrast, social professionals are generally less involved in direct coercive interventions and are more likely to advocate patient autonomy and non-coercive therapeutic approaches [79]. Moreover, greater exposure of nursing staff to workplace violence in mental health [80] may further support the belief that coercion is an essential safeguard [28,81].

The work setting also emerged as a significant factor influencing attitudes toward coercion, which is consistent with previous research [64,65]. Participants working in residential settings reported the highest negative and lowest pragmatic attitudes, whereas those employed in acute psychiatric units exhibited stronger pragmatic and positive attitudes. These differences likely reflect distinct clinical contexts and patient populations encountered across settings. Acute inpatient units are typically characterized by high-intensity care and frequent management of individuals in crisis, including those experiencing a first episode of psychosis, a circumstance associated with an increased risk of aggression [82]. In such environments, staff are more exposed to aggression, which heightens the likelihood of encountering and applying coercive measures [28,83]. Repeated exposure may foster a sense of procedural familiarity and perceived legitimacy, reinforcing more accepting views [39,64,66].

However, beyond individual characteristics, the use of coercion should be understood as a socially embedded practice shaped by the cultural norms and institutional contexts of each facility [27,37,74]. Psychiatric units function as microcultures, where implicit and explicit rules guide how staff are expected to respond to aggression and safety challenges. These social expectations influence not only individual decision-making but also collective responses and team dynamics [17,18,31,35,46]. Thus, attitudes toward coercion may also reflect shared norms and learned behaviors within settings and teams [27].

### 4.1. Implications for Practice and Policy

The findings of this study highlight the complex and context-sensitive nature of MHCWs’ attitudes toward coercion, offering important implications for clinical practice and mental health policies. Targeted interventions, such as structured training programs and ethical debriefing sessions, should be prioritized to reduce reliance on coercion [84,85] and promote attitudinal change [34]. These efforts should focus on the roles and settings in which favorable attitudes toward coercion persist. At the policy level, advancing a humane, rights-based mental health system requires systematic monitoring of the prevalence and patterns of coercive practices through standardized methods [86] and the promotion of evidence-based alternatives such as open-door policies and security technologies [87,88]. Education and ongoing professional development could also be a key. In the Italian context, national efforts to establish postgraduate specialization in mental health nursing could help cultivate rights-oriented and rights-based professional identities among frontline staff. Finally, organizational strategies, such as appropriate staff-to-patient ratios, providing continuous support, and the creation of therapeutic environments, are essential to both reducing the use of coercion and addressing the moral conflict experienced by MHCWs [20,21,23,89].

### 4.2. Limitations and Future Research

To our knowledge, this is the first and largest empirical study to examine MHCWs’ attitudes toward coercion and its associated factors in the Italian context. However, this study has some limitations that must be acknowledged. First, data were collected between May and December 2023. Although no major policy or structural changes have occurred since then, the findings may not entirely capture current practices. Second, the final sample was slightly below the threshold required for statistical generalizability, and the use of a convenience sample from a single mental health department may further limit the external validity of the results. Third, the cross-sectional design precludes causal inferences; thus, it remains unclear whether attitudes lead to coercive practices. Fourth, the small sample sizes of some subgroups (i.e., other professionals) may have reduced the reliability of subgroup estimates. In addition, potentially relevant variables, such as educational background, exposure to patient aggression, and prior training in coercion alternatives, were not assessed. Lastly, the study did not include qualitative data and narratives about personal experiences with coercion.

Future research should adopt longitudinal and mixed-methods approaches to investigate how attitudes toward coercion evolve over time and whether they influence clinical behavior. In particular, qualitative studies could offer deeper insights into the ethical reasoning and institutional cultures that shape these attitudes. Furthermore, exploring the implicit and explicit rules within psychiatric units, as well as the social processes that influence professionals’ responses, may reveal important contextual factors associated with coercive practices. Finally, incorporating a broader set of variables and extending the research to a national level would enhance generalizability and better inform clinical guidelines and policy development.

## 5. Conclusions

This study provides an initial empirical contribution to the understanding of Italian MHCWs’ attitudes toward coercion and their association with sociodemographic and professional characteristics. Participants expressed moderately negative attitudes, with a pragmatic view of coercion as necessary for safety, but limited endorsement of its therapeutic role, reflecting the ambivalence reported in the international literature. Significant differences by age, sex, professional role, and work setting underscored the influence of both individual and contextual factors. These findings highlight the need for targeted, role- and setting-specific strategies to challenge the normalization of coercion and promote more ethical, rights-based, and person-centered practices. This study offers a foundation for shaping national policy, workforce education, and quality improvement initiatives in Italy. Future research should investigate the relationship between attitudes and actual clinical behavior, incorporate qualitative perspectives of stakeholders, and expand across broader settings to support reform efforts at both national and international levels.

## Figures and Tables

**Table 1:** Sociodemographic and professional characteristics of the mental healthcare workers.

		n	%	Mean	SD (±)	Min	Max	Median
Sex	Male	202	56.7					
	Female	154	43.3					
Age (years)	<40	63	17.7	51.08	10.59	23	66	53.5
	40–49	66	18.5					
	50–59	132	37.1					
	≥60	95	26.7					
Working experience (years)				22.61	12.81	1	43	27.5
Working experience in mental health (years)	<5	135	37.9	13.74	12.14	1	37	11.0
	5–20	114	32.0					
	>20	107	30.1					
Professional role	Nurses	179	50.3					
	Nursing aides	100	28.1					
	Psychiatrists	52	14.6					
	Other healthcare workers ^1^	25	7.0					
Work unit	General Hospital Psychiatric Units	185	52.0					
	Community Mental Health Centers	89	25.0					
	Residential Facilities	77	21.6					
	Day Care Facilities	5	1.4					

^1^ Social workers, psychologists, pedagogists, psychiatric rehabilitation technicians, animators, educators, physiotherapists, and sociologists.

**Table 2:** Results of the Staff Attitude to Coercion Scale (SACS) describing negative, pragmatic, and positive attitudes toward coercion.

	Mean	SD (±)
Negative attitudes (Coercion as offensive)	3.06	0.79
Use of coercion can harm the therapeutic relationship	2.94	1.14
Too much coercion is used in treatment	2.42	1.00
Scarce resources lead to more use of coercion	3.40	1.12
Coercion could have been much reduced, giving more time and personal contact	3.49	1.13
Pragmatic attitudes (Coercion as care and security)	3.33	0.67
Use of coercion is necessary as protection in dangerous situations	3.93	0.96
For security reasons, coercion must sometimes be used	3.32	1.21
Use of coercion is a declaration of failure on the part of the mental health services	3.40	1.22
Coercion may represent care and protection	3.29	1.11
Coercion may prevent the development of a dangerous situation	3.77	1.07
Coercion violates the patients’ integrity	2.45	0.99
For severely ill patients, coercion may represent safety	3.14	1.15
Positive attitudes (Coercion as treatment)	2.70	0.71
More coercion should be used in treatment	2.00	0.97
Patients without insight require use of coercion	2.55	1.08
Use of coercion is necessary toward dangerous and aggressive patients	4.04	0.99
Regressive patients require use of coercion	2.21	1.00

**Table 3:** Correlation between age, working experience, and working experience in mental health with negative, pragmatic, and positive attitudes toward coercion.

	Negative Attitudes	Pragmatic Attitudes	Positive Attitudes
Age (years)	0.071	−0.128 *	−0.051
Working experience (years)	0	−0.092	−0.045
Working experience in mental health (years)	−0.025	−0.055	0.040

Table values refer to the correlation coefficient (r). * = correlation significant at *p* ≤ 0.05.

**Table 4:** Negative, pragmatic, and positive attitudes toward coercion across sex, age, working experience in mental health, professional roles, and work settings.

	Negative Attitudes (Mean, ±SD)	Pragmatic Attitudes (Mean, ±SD)	Positive Attitudes (Mean, ±SD)
Sex	*p* = 0.256	*p* = 0.310	*p* = 0.001 *
Male	3.02 (0.81)	3.36 (0.69)	2.80 (0.74)
Female	3.11 (0.77)	3.29 (0.65)	2.56 (0.64)
Age (years)	*p* = 0.087	*p* = 0.012 *	*p* = 0.419
<40	3.08 (0.90)	3.34 (0.73)	2.71 (0.69)
40–49	3.05 (0.75)	3.45 (0.67)	2.66 (0.69)
50–59	2.94 (0.71)	3.39 (0.58)	2.77 (0.70)
≥60	3.22 (0.84)	3.14 (0.72)	2.62 (0.72)
Working experience in mental health (years)	*p* = 0.188	*p* = 0.575	*p* = 0.990
<5	3.10 (0.83)	3.37 (0.68)	2.71 (0.75)
5–20	2.95 (0.74)	3.31 (0.62)	2.69 (0.67)
>20	3.13 (0.80)	3.29 (0.72)	2.69 (0.69)
Professional role	*p* < 0.001 *	*p* < 0.001 *	*p* < 0.001 *
Nurses	2.93 (0.78)	3.39 (0.62)	2.70 (0.65)
Nursing aides	3.02 (0.72)	3.38 (0.60)	3.00 (0.75)
Psychiatrists	3.18 (0.74)	3.30 (0.80)	2.40 (0.56)
Other healthcare workers ^1^	3.90 (0.76)	2.72 (0.73)	2.11 (0.54)
Work setting	*p* < 0.001 *	*p* < 0.001 *	*p* = 0.049 *
Acute	2.78 (0.71)	3.50 (0.58)	2.78 (0.70)
Community	3.21 (0.70)	3.28 (0.70)	2.56 (0.60)
Residential ^2^	3.53 (0.80)	3.00 (0.71)	2.68 (0.80)

* = significant difference in means across groups; ^1^ = Social workers, psychologists, pedagogists, psychiatric rehabilitation technicians, animators, educators, physiotherapists, and sociologists; ^2^ = Residential Facilities and Day Care Facilities.

## Data Availability

The data presented in this study are available on request from the corresponding author.

## Abbreviations

The following abbreviations are used in this manuscript:

MHCW	Mental Healthcare Worker
SACS	Staff Attitude to Coercion Scale
STROBE	Strengthening the Reporting of Observational Studies in Epidemiology
CVR	Content Validity Ratio
I-CVI	Item-level Content Validity Index
S-CVI	Scale-level Content Validity Index
M	Mean
SD	Standard Deviation
CMHC	Community Mental Health Center
GHPU	General Hospital Psychiatric Unit
DCF	Day Care Facility
RF	Residential Facility
SPSS	Statistical Package for the Social Sciences

## Appendix A

**Table A1:** STROBE Statement—Checklist of items that should be included in reports of cross-sectional studies.

	Item No	Recommendation	Page No
Title and abstract	1	(a) Indicate the study’s design with a commonly used term in the title or the abstract	1
		(b) Provide in the abstract an informative and balanced summary of what was done and what was found	1
Introduction			
Background/rationale	2	Explain the scientific background and rationale for the investigation being reported	2
Objectives	3	State specific objectives, including any prespecified hypotheses	3
Methods			
Study design	4	Present key elements of study design early in the paper	3
Setting	5	Describe the setting, locations, and relevant dates, including periods of recruitment, exposure, follow-up, and data collection	3
Participants	6	(a) Give the eligibility criteria, and the sources and methods of selection of participants	3
Variables	7	Clearly define all outcomes, exposures, predictors, potential confounders, and effect modifiers. Give diagnostic criteria, if applicable	3
Data sources/measurement	8 *	For each variable of interest, give sources of data and details of methods of assessment (measurement). Describe comparability of assessment methods if there is more than one group	3, 4
Bias	9	Describe any efforts to address potential sources of bias	4
Study size	10	Explain how the study size was arrived at	3
Quantitative variables	11	Explain how quantitative variables were handled in the analyses. If applicable, describe which groupings were chosen and why	4
Statistical methods	12	(a) Describe all statistical methods, including those used to control for confounding	4
		(b) Describe any methods used to examine subgroups and interactions	4
		(c) Explain how missing data were addressed	4
		(d) If applicable, describe analytical methods taking account of sampling strategy	4
		(e) Describe any sensitivity analyses	4
Results			
Participants	13 *	(a) Report numbers of individuals at each stage of study—e.g., numbers potentially eligible, examined for eligibility, confirmed eligible, included in the study, completing follow-up, and analyzed	4, 5
		(b) Give reasons for non-participation at each stage	-
		(c) Consider use of a flow diagram	-
Descriptive data	14 *	(a) Give characteristics of study participants (e.g., demographic, clinical, social) and information on exposures and potential confounders	4, 5
		(b) Indicate number of participants with missing data for each variable of interest	4, 5
Outcome data	15 *	Report numbers of outcome events or summary measures	5, 6
Main results	16	(a) Give unadjusted estimates and, if applicable, confounder-adjusted estimates and their precision (e.g., 95% confidence interval). Make clear which confounders were adjusted for and why they were included	-
		(b) Report category boundaries when continuous variables were categorized	5, 6
		(c) If relevant, consider translating estimates of relative risk into absolute risk for a meaningful time period	-
Other analyses	17	Report other analyses done—e.g., analyses of subgroups and interactions, and sensitivity analyses	6, 7
Discussion			
Key results	18	Summarize key results with reference to study objectives	7–9
Limitations	19	Discuss limitations of the study, taking into account sources of potential bias or imprecision. Discuss both direction and magnitude of any potential bias	9
Interpretation	20	Give a cautious overall interpretation of results considering objectives, limitations, multiplicity of analyses, results from similar studies, and other relevant evidence	7–9
Generalizability	21	Discuss the generalizability (external validity) of the study results	7–9
Other information			
Funding	22	Give the source of funding and the role of the funders for the present study and, if applicable, for the original study on which the present article is based	10

* Give information separately for exposed and unexposed groups.
